# Individualized treatment for allergic rhinitis based on key nasal clinical manifestations combined with histamine and leukotriene D4 levels^☆^

**DOI:** 10.1016/j.bjorl.2018.09.007

**Published:** 2018-10-24

**Authors:** Congxiang Shen, Fang Chen, Huigang Wang, Xinyu Zhang, Guanxue Li, Zhong Wen

**Affiliations:** Department of Otorhinolaryngology Head and Neck Surgery, Southern Medical University, Zhujiang Hospital, Guangzhou, China

**Keywords:** Allergic rhinitis, Clinical typing, Individualized treatment, Histamine, Leukotrienes, Rinite alérgica, Tipificação clínica, Tratamento individualizado, Histamina, Leucotrienos

## Abstract

**Introduction:**

The types of allergic rhinitis are roughly classified based on the causative antigens, disease types, predilection time, and symptom severity.

**Objective:**

To examine the clinical typing and individualized treatment approach for allergic rhinitis and to determine the optimal treatment method for this disease using various drug combination therapies.

**Methods:**

A total of 108 participants with allergic rhinitis were divided into three groups based on symptoms. Subsequently, each group was further categorized into four subgroups based on the medications received. The efficacy of the treatments was evaluated using the visual analog scale *VAS* scores of the total and individual nasal symptoms, decline index of the symptom score, histamine and leukotriene levels, and mRNA and protein expression levels of histamine 1 and cysteinyl leukotriene 1 receptors.

**Results:**

Loratadine + mometasone furoate and loratadine + mometasone furoate + montelukast significantly improved the sneezing symptom and reduced the histamine levels compared with the other combination therapies (*p *< 0.05). Meanwhile, montelukast + mometasone furoate and montelukast + mometasone furoate + loratadine considerably improved the nasal obstruction symptom and decreased the leukotriene D4 levels compared with the other combination therapies (*p* < 0.05).

**Conclusion:**

Clinical symptom evaluation combined with experimental detection of histamine and leukotriene levels can be an objective and accurate method to clinically classify the allergic rhinitis types. Furthermore, individualized treatment based on allergic rhinitis classification can result in a good treatment efficacy.

## Resumo

**Introdução:**

A rinite alérgica é basicamente classificada de acordo com os antígenos causadores, tipos de doença, peridiocidade e gravidade dos sintomas.

**Objetivo:**

Avaliar os tipos clínicos e a abordagem terapêutica individualizada para cada tipo de rinite alérgica e determinar o método de tratamento ideal utilizando várias terapias de combinação de fármacos.

**Método:**

Um total de 108 participantes com rinite alérgica foram divididos em três grupos com base nos sintomas. Posteriormente, cada grupo foi subsequentemente categorizado em quatro subgrupos com base nos medicamentos recebidos. A eficácia dos tratamentos foi avaliada utilizando os escores da escala visual analógica EVA dos sintomas nasais totais e individualmente, índice de declínio do escore de sintomas, níveis de histamina e leucotrienos e níveis de expressão de mRNA e proteína dos receptores de histamina 1 e cisteinil-leucotrieno 1.

**Resultados:**

As associações entre loratadina + furoato de mometasona, assim como a de loratadina + furoato de mometasona + montelucaste melhoraram significativamente o sintoma de espirros e reduziram os níveis de histamina em comparação às outras terapias combinadas (*p* < 0,05). Por outro lado, a associação montelucaste + furoato de mometasona, assim como a associação montelucaste + furoato de mometasone + loratadina melhoraram consideravelmente o sintoma de obstrução nasal e diminuíram os níveis de leucotrieno D4 em comparação com as outras combinações (*p* < 0,05).

**Conclusão:**

A avaliação clínica dos sintomas combinada com a detecção experimental dos níveis de histamina e leucotrieno pode ser um método objetivo e preciso para classificar clinicamente os tipos de rinite alérgica. Além disso, o tratamento individualizado baseado na classificação da rinite alérgica pode resultar no aumento da eficácia do tratamento.

## Introduction

The development of allergic rhinitis (AR) mainly involves a T-helper Type 2 pattern of mucosal cell hyperfunction,^1^, ^2^ which results in chronic inflammation of the nasal mucosa caused by the release of a series of inflammatory mediators, primarily histamine and leukotriene.^3^ Recent research demonstrated that the release of leukotriene occurs in both the early and late phases of hypersensitivity responses.^4^ This finding led to changes in the treatment concept of AR, in which the effects of antileukotriene treatment were found to be comparable to those of antihistamines and intranasal corticosteroids. In general, effective management for AR is comprised of allergen avoidance, increased patient awareness and education, pharmacotherapy, and allergen-specific immunotherapy, among others.^5^ Currently, the medications used to clinically control AR are classified into three major types: second-generation antihistamines, antileukotrienes, and intranasal corticosteroids. Various investigations and meta-analyses analyzed the effect of different combinations using these three drugs on AR, and their results showed that drug combinations were more effective than single drug therapy.^6^, ^7^, ^8^, ^9^, ^10^ However, the development and evaluation of these treatment methods were still based on the participants’ subjective reports of nasal symptoms and quality of life. Additionally, few objective laboratory indexes were used in combination with the participants’ subjective appraisals in some research. In the present study, we aimed to classify the AR patients into several groups depending on the primary nasal symptoms and quantitative detection of the levels of inflammatory mediators (histamine and leukotriene D4) released during hypersensitivity reactions in the peripheral blood and nasal secretions to objectively present the practical in vivo and in vitro responses of individual participants. Additionally, we aimed to design various drug combination treatment methods and determine the optimal treatment methods through comparison of pre- and post-therapy improvements in nasal symptoms and changes in histamine and leukotriene D4 levels, as well as mRNA and protein expression levels of Histamine 1 (H1) and Cysteinyl Leukotriene 1 (CysLT1) receptors. The findings of this study will serve as a basis for the exploration of new approaches for individualized AR treatment.

## Materials and methods

### Study population and clinical parameters

Participants with AR were recruited from the outpatient department of Zhujiang Hospital, Southern Medical University (Guangzhou, China) from January 2014 to June 2015. The participants were selected through medical history and allergic symptom assessments based on the inclusion and exclusion criteria, which are patterned after a previously reported guideline.^11^

The inclusion criteria are as follows: (1) age 18–65 years, with a history of AR for at least one year; (2) at least three of the four major symptoms (sneezing, clear nasal discharge flow, nasal obstruction, and nasal itching), with these symptoms having >0.5–1 h one-day cumulative attack time for participants with Perennial AR (PAR); (3) nasal mucosa (or even eyelid) edema and obstruction; and (4) positive reaction to at least one of the allergen skin prick tests, with a skin allergy test index score that is not more than ++ or +++.

Meanwhile, the exclusion criteria include the following: (1) existence of respiratory tract infection and infectious suborbital nasosinusitis; (2) presence of allergic asthma, with asthmatic attack during the last 5 years; (3) existence of non-AR (a condition in which the causative allergen was not confirmed, e.g., vasomotor rhinitis); (4) presence of severe nasal septum deviation or nasal polyps; (5) <4 weeks of antihistamine (e.g., Claritin) and corticosteroid drug discontinuation; (6) <6 weeks of astemizole discontinuation and <1 week withdrawal time for other antihistamine drugs; (7) <1 week withdrawal time for nasal suction antihistamines, glucocorticoid nasal sprays, or nose drop drugs; and (8) use of macrolide antibiotics and/or imidazole antifungal agents.

The study protocol was approved by the Ethics Committee of Zhujiang Hospital, Southern Medical University, and all participants provided written informed consent. Furthermore, this study was conducted in accordance with the regulations of the Clinical Research Ethics Committee and World Medical Association and the principles of the Declaration Helsinki (Clinical Trial Registration number: 2013-EBYHK-004).

### Study population characteristics and treatment grouping

A total of 108 participants (62 men and 46 women) with a mean age of 37 years were included in this study. Thirty-six participants (20 men and 16 women) with a mean age of 36 years underwent physical assessment in the physical examination center of Zhujiang Hospital, who were then enrolled as healthy controls.

**Figure 1:**
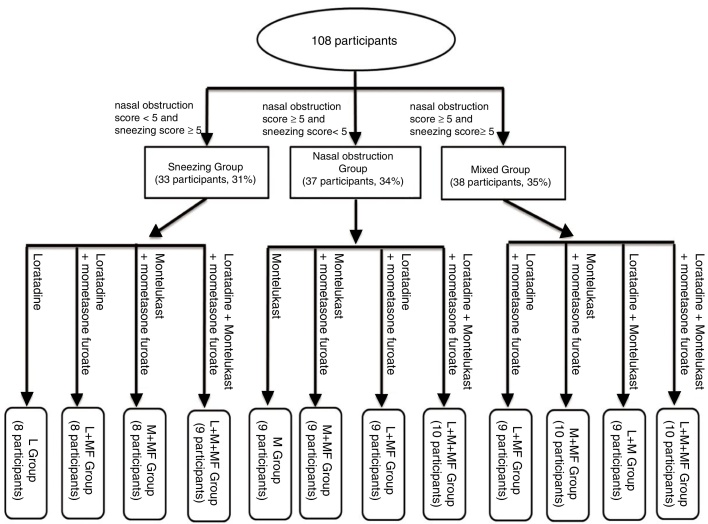
Schematic illustration of treatment grouping of AR participants.

The sneezing and nasal obstruction scores of all participants were obtained, which were used for treatment grouping, using the Visual Analogue Scale (VAS) scores that are based on symptom severity (Table 1). As shown in Fig. 1, all participants were classified into three groups based on the symptoms’ VAS score combined with the histamine and leukotriene D4 levels. The 108 participants were divided into sneezing (33 participants, 31%), nasal obstruction (37 participants, 34%), and mixed (38 participants, 35%) groups. Subsequently, the three symptom groups were further divided into four subgroups based on the medications they received. In this study, loratadine, montelukast, and mometasone furoate were used to treat the patients with AR. The participants randomly received 10 mg loratadine and montelukast (one dose per day) and 50 μg mometasone furoate (one press for each nostril and one dose per day).

**Table 1:** Single nasal symptom VAS score based on severity of symptoms.

Symptom	Domain	Scale
Sneezing^a^	No symptom	0 (no symptom)
	3–5	1–2 (mild)
	6–10	3–6 (moderate)
	≥11	7–10 (severe)

Nasal obstruction	No symptom	0 (no symptom)
	Awareness but not troubled	1–2 (mild)
	Intermittent or alternation	3–6 (moderate)
	Almost all mouth breathing	7–10 (severe)

a The daily number of continuous sneezing.

### Determination of histamine and leukotriene D4 levels

The venous blood and nasal secretions of the participants with AR and those of the healthy controls were extracted to determine the histamine and leukotriene D4 levels using double-antibody sandwich enzyme-linked immunosorbent assay. The enzyme immunoassay kit was purchased from Shanghai BlueGene Biotech Co., Ltd. (Shanghai, China), and the assays were performed in accordance with the manufacturer's instructions using a Synergy HT Multimode Reader (BioTek, Winooski, VT, USA).

### Gene and protein expression of H1 and CysLT1 receptors

The turbinate mucosal tissues of participants with AR and those who underwent nasal septum surgery, who were included as healthy controls, were examined to characterize the gene and protein expressions of H1 and CysLT1 receptors. A real-time fluorescent quantitative Polymerase Chain Reaction (PCR) was performed to analyze the mRNA expression of H1 and CysLT1 receptors. All the oligonucleotides purified through high-performance liquid chromatography were chemically synthesized by Invitrogen Biotechnology Co. Ltd. (Shanghai, China). The primers F1 (CCTGGATACCGCAGCTAGGA) and R1 (GCGGCGCAATACGAATGCCCC) were used for 18SrRNA amplification (GenBank ID: NR 003286) as an internal reference. Meanwhile, the primers F2 (TACGGAGTGAGCGGAAGC) and R2 (GCAGGTAGAGGATGTTCATAGG) were used for HRH1 amplification (GenBank ID: NM 000861.3). Furthermore, the primers F3 (GCCATGAGCTTTTTCCGGTG) and R3 (TGATTGTCTTGTGGGGGCTC) were used for CysLT1 amplification (GenBank ID: AF 119711.1). The reverse transcription reaction system for cDNA synthesis was prepared based on the manufacturer's instruction for the use of GoScript™ Reverse Transcription System. Quantitative Polymerase Chain Reaction (qPCR) was performed using ABI7500 real-time qPCR Thermocycle Instrument (ABI, Vernon, CA, USA), with cDNA as a template of FastStart Universal SYBR Green Master (Rox) (Roche, Indianapolis, IN, USA) and β-actin as a control, in accordance with the manufacturer's instruction. The average threshold Cycle (Ct) values for the three PCR reactions were determined and calculated as fold induction over the control samples using the comparative ΔCt method to calculate the changes in gene expression level.

The protein expression of H1 and CysLT1 receptors was examined through Western blot assay. Additionally, a semiquantitative chemiluminescence (CL) analysis was conducted using the Image Lab software after a grayscale scanning of the X-ray film. The average CL for three cases were determined and calculated as the relative CL over the control samples to calculate the changes in the protein expression level. The protein expression of H1 and CysLT1 receptors were quantified through comparisons with β-actin and γ-tubulin, respectively.

### Clinical treatment evaluation

The Total Nasal Symptom Score (TNSS) was calculated as the sum of the scores of the four nasal symptoms (rhinorrhea, nasal itching, nasal obstruction, and sneezing), which were assessed using a four-point Likert scale based on symptom severity (Table 2), in accordance with the protocol of a previous report.^11^ The single nasal symptom scores were recorded through outpatient service and telephone surveys of participants, which were conducted on the consultation day and 2, 4, and 8 weeks after medication therapy. The Total Nasal Symptom Score Reducing Index (TSSRI), sneezing score Reducing Index (RI), and nasal obstruction score RI were used to evaluate the efficacy of the clinical treatment, in which RI = (pre-treatment score − post-treatment score)/pre-treatment score. Additionally, the histamine and leukotriene D4 levels in the peripheral blood and nasal secretions, together with the changes in the mRNA and protein expression levels of H1 and CysLT1 receptors in the turbinate mucosa tissues were also used to assess the clinical curative effect.

**Table 2:** Assessment scale based on severity of symptoms.

Symptom	Domain	Scale
Rhinorrhea^a^	No symptom	0
	3–5	1
	6–10	2
	≥11	3

Sneezing^b^	No symptom	0
	3–5	1
	6–10	2
	≥11	3

Nasal itching	No symptom	0
	Intermittent	1
	Formication but can stand it	2
	Formication and cannot stand it	3

Nasal obstruction	No symptom	0
	Awareness but not troubled	1
	Intermittent or alternation	2
	Almost all mouth breathing	3

a The daily number of blowing nose.

b The daily number of continuous sneezing.

### Statistical analysis

The correlations between the single nasal symptom score (sneezing and nasal obstruction scores) and histamine and leukotriene D4 levels in the peripheral blood and nasal secretions were assessed using Pearson's correlation test. An independent sample *t* test was conducted to determine the RI of the single nasal symptom score and histamine and leukotriene D4 levels before and after treatment and evaluate the validity of the pre- and post-medication effects for each group. Furthermore, a paired sample *t* test was performed to calculate the TNSSRI of the AR participants after 2, 4, and 8 weeks of treatment. A *p*-value < 0.05 was considered statistically significant. All data were presented as Ā ± S, where Ā and S refer to the mean value and standard deviation of the mean, respectively. All statistical analyses were performed using SPSS version 19.0 (IBM Co., Armonk, NY, USA).

## Results

### Clinical characteristics of AR participants from the three symptom groups before treatment

**Figure 2:**
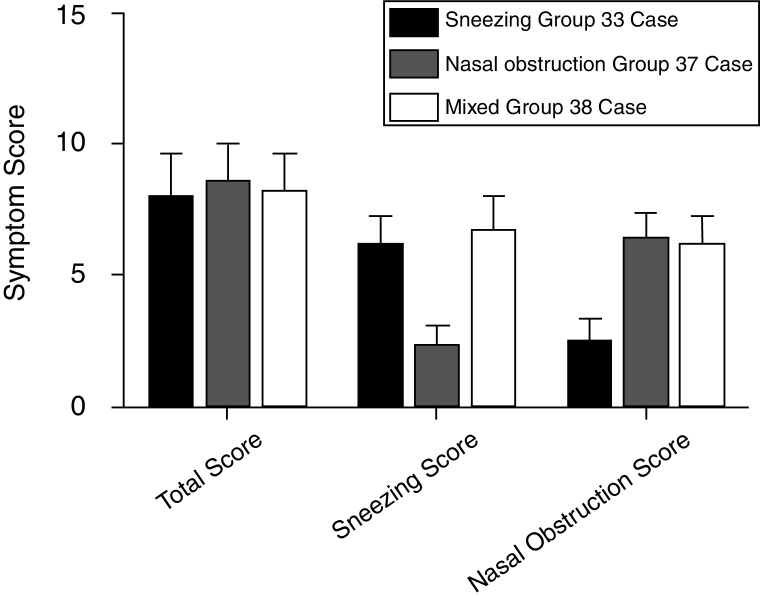
Total nasal symptom score and single nasal symptom score of AR participants from sneezing group (*n* = 33), nasal group (*n* = 37) and mixed group (*n* = 38) before treatment.

Fig. 2 presents the AR clinical status of the participants from the sneezing (*n* = 33), nasal (*n* = 37), and mixed (*n* = 38) groups before treatment based on the TNSS and sneezing and nasal obstruction scores. The TNSS parameters of the three symptom groups were not statistically significant (*p* > 0.05). The sneezing and mixed groups showed significantly higher sneezing scores than the nasal obstruction group (*p* < 0.05). The nasal obstruction and mixed groups had significantly higher nasal obstruction scores than the sneezing group (*p* < 0.05). Meanwhile, Table 3 displays the mean histamine and leukotriene D4 levels in the peripheral blood and nasal secretions of the AR participants from the three groups. The TNSS and histamine and leukotriene D4 levels of the three symptom groups showed markedly positive correlations (*p* < 0.001). Table 4 presents the correlations between the single nasal symptom score and histamine and leukotriene D4 levels in the peripheral blood and nasal secretions. A positive relationship was observed between the sneezing VAS scores and histamine levels in the peripheral blood and nasal secretions of the AR participants from the sneezing, nasal obstruction, and mixed groups (*r* = 0.43/0.43, 0.60/0.61, and 0.83/0.82, respectively; *p* < 0.05). Similarly, highly positive correlations were also found between the nasal obstruction VAS score and leukotriene D4 levels of the sneezing, nasal obstruction, and mixed groups (*r* = 0.54/0.50, 0.56/0.55, and 0.61/0.60, respectively; *p* < 0.05).

**Table 3:** Histamine and leukotriene D4 levels in peripheral blood and nasal secretions of AR participants from sneezing group (*n* = 33), nasal group (*n* = 37) and mixed group (*n* = 38) before treatment and correlations of total nasal symptom score and histamine and leukotriene D4 levels in peripheral blood and nasal secretions.

Group	Number of patients	Histamine levels (ng/mL)		Leukotriene D4 levels (ng/mL)		Correlation (*r*)	
		In peripheral blood	In nasal secretions	In peripheral blood	In nasal secretions	His^a^	LtD4^b^
Sneezing group	33	8.61 ± 3.05	5.20 ± 1.90	0.225 ± 0.098	0.135 ± 0.068	0.98	0.98
Nasal congestion group	37	4.76 ± 2.54	2.88 ± 1.50	0.444 ± 0.190	0.266 ± 0.113	0.99	0.97
Mixed group	38	11.82 ± 3.04	7.11 ± 1.88	0.495 ± 0.143	0.297 ± 0.107	0.99	0.99

a *p* < 0.001.

b *p* < 0.001.

**Table 4:** Correlations between the single nasal symptom score and histamine and leukotriene D4 levels in the peripheral blood and nasal secretions.

Group	Nasal symptom	*r* (*p*)			
		Histamine levels		Leukotriene D4 levels	
		In peripheral blood	In nasal secretions	In peripheral blood	In nasal secretions
Sneezing group	Sneezing	0.43 (0.01)	0.43 (0.01)	0.05 (0.74)	0.13 (0.45)
	Nasal obstruction	0.17 (0.32)	0.19 (0.28)	0.54 (0.002)	0.50 (0.002)

Nasal congestion group	Sneezing	0.60 (0.00)	0.61 (0.00)	0.01 (0.94)	−0.10 (0.57)
	Nasal obstruction	−0.21 (0.21)	−0.27 (0.11)	0.56 (0.00)	0.55 (0.00)

Mixed group	Sneezing	0.83 (0.00)	0.82 (0.00)	−0.06 (0.75)	0.18 (0.30)
	Nasal obstruction	−0.11 (0.53)	0.39 (0.02)	0.61 (0.00)	0.60 (0.00)

### Clinical outcomes of AR participants after medication therapy based on the symptom scores and histamine and leukotriene D4 levels

**Figure 3:**
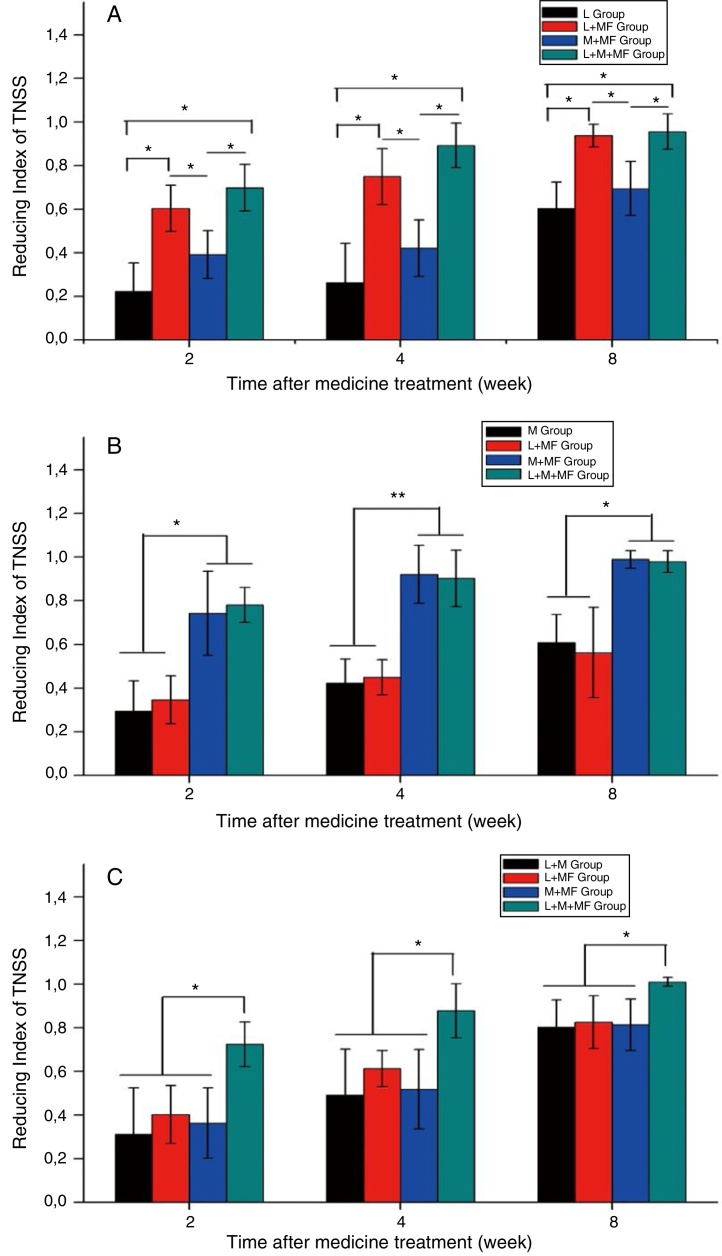
Total Nasal Symptom Score Reducing Index (TNSSRI) of AR participants for (A) sneezing group, (B) nasal obstruction group and (C) mixed group after 2, 4 and 8 weeks of medication.

Fig. 3 displays the TNSSRI after treatment of the sneezing, nasal obstruction, and mixed groups. For the sneezing group (Fig. 3A), the loratadine + mometasone furoate and loratadine + montelukast + mometasone furoate combination therapies had significantly lower TNSS than the loratadine monotherapy and montelukast + mometasone furoate combination therapy after a specific medication administration period (*p* < 0.05). For the nasal obstruction group (Fig. 3B), both the montelukast + mometasone furoate and loratadine + montelukast + mometasone furoate combination therapies had significantly lower TNSS than the montelukast monotherapy and loratadine + mometasone furoate combination therapy (*p* < 0.05). Meanwhile, for the mixed group (Fig. 3C), the loratadine + montelukast + mometasone furoate combination therapy had significantly lower TNSS than the loratadine + montelukast, loratadine + mometasone furoate, and montelukast + mometasone furoate combination therapies (*p* < 0.05).

**Figure 4:**
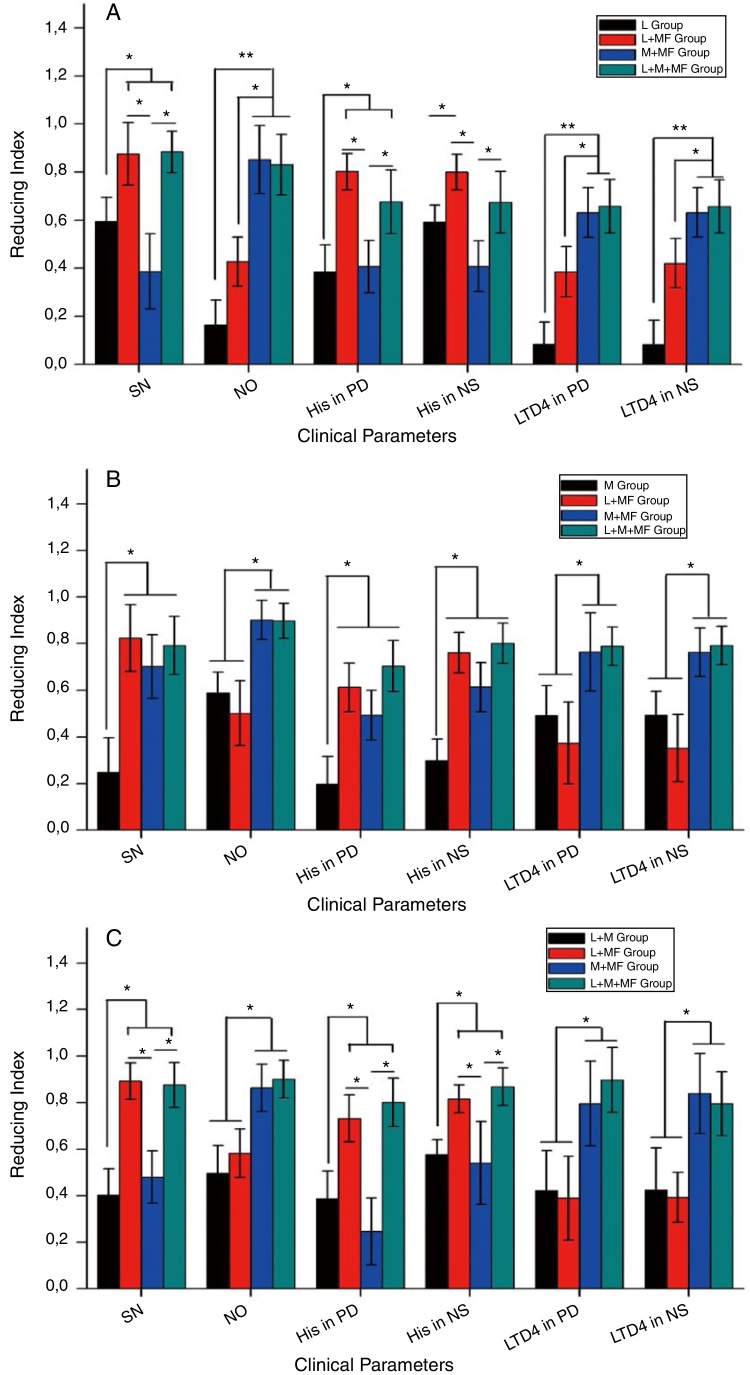
Reducing index of sneezing score, nasal obstruction score and levels of histamine and leukotriene D4 in peripheral blood and nasal secretions of AR participants for (A) sneezing group, (B) nasal obstruction group and (C) mixed group after 4 weeks of medication. SN, stands for sneezing score; NO, stands for nasal obstruction score; His in PD, stands for level of histamine in peripheral blood, His in NS, stands for level of histamine in nasal secretions, LTD4 in PD, stands for level of leukotriene D4 in peripheral blood; LTD4 in NS, stands for level of leukotriene D4 in nasal secretions.

Fig. 4 shows the RI of the various clinical parameters, such as sneezing and nasal obstruction scores and histamine and leukotriene D4 levels in the peripheral blood and nasal secretions, after treatment for the sneezing, nasal obstruction, and mixed groups. Briefly, for the sneezing group (Fig. 4A), loratadine + mometasone furoate and loratadine + montelukast + mometasone furoate combination therapies significantly improved the sneezing symptom and reduced the histamine levels in the peripheral blood and nasal secretions compared with loratadine monotherapy (*p* < 0.05) and montelukast + mometasone furoate combination therapy (*p* < 0.05). Furthermore, both montelukast + mometasone furoate and loratadine + montelukast + mometasone furoate combination therapies significantly improved the nasal obstruction symptom and decreased the leukotriene D4 levels in the peripheral blood and nasal secretions compared with loratadine monotherapy (*p* < 0.01) and loratadine + mometasone furoate combination therapy (*p* < 0.05). For the nasal obstruction group (Fig. 4B), loratadine + mometasone furoate, montelukast + mometasone furoate, and loratadine + montelukast + mometasone furoate combination therapies significantly improved the sneezing symptom and reduced the histamine levels in the peripheral blood and nasal secretions compared with loratadine monotherapy (*p* < 0.05). Moreover, both montelukast + mometasone furoate and loratadine + montelukast + mometasone furoate combination therapies significantly improved the nasal obstruction symptom and lowered the leukotriene D4 levels in the peripheral blood and nasal secretions compared with montelukast monotherapy (*p* < 0.05) and loratadine + mometasone furoate combination therapy (*p* < 0.05). For the mixed group (Fig. 4C), both loratadine + mometasone furoate and loratadine + montelukast + mometasone furoate combination therapies significantly improved the sneezing symptom and reduced the histamine levels in the peripheral blood and nasal secretions compared with loratadine + montelukast and montelukast + mometasone furoate combination therapies (*p* < 0.05). Additionally, both montelukast + mometasone furoate and loratadine + montelukast + mometasone furoate combination therapies significantly improved the nasal obstruction symptom and decreased the leukotriene D4 levels in the peripheral blood and nasal secretions compared with loratadine + montelukast and loratadine + mometasone furoate combination therapies (*p* < 0.05).

### Gene and protein expressions of H1 and CysLT1 receptors

**Figure 5:**
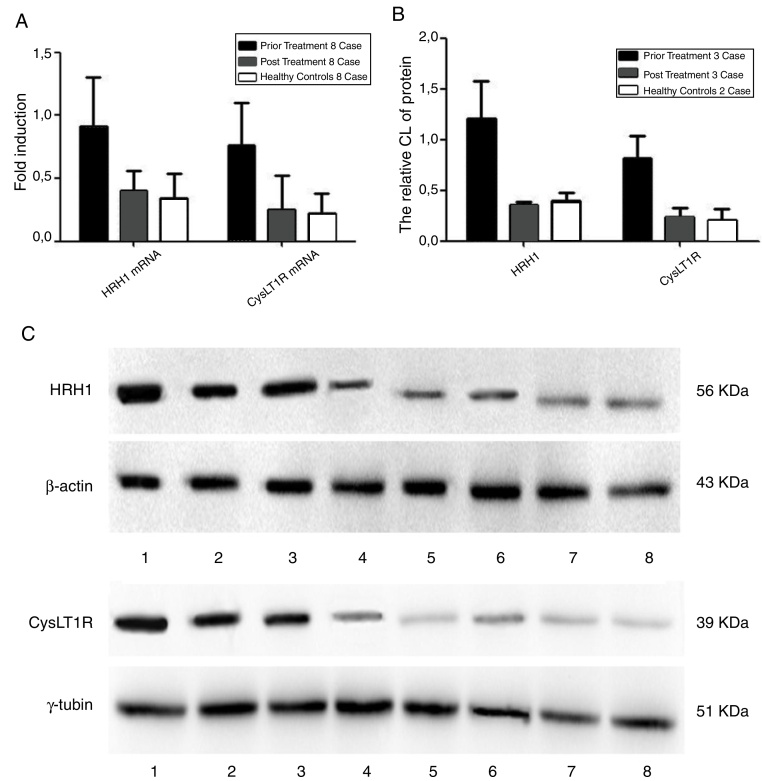
Detection of gene expression and protein expression levels of histamine H1 and leukotriene CysLT1 receptors of AR participants before and after medication. (A) Quantification of mRNA levels of histamine H1 and leukotriene CysLT1 receptors analyzed by qPCR. (B) Quantification of protein levels of histamine H1 and leukotriene CysLT1 receptors analyzed by Western blot. (C) Western blot images of histamine H1, leukotriene CysLT1 and control proteins. Prior treatment: Lane 1, 2, 3; post treatment: Lanes 4, 5, 6, and healthy control: Lanes 7, 8. β-Actin and γ-tublin were respectively used as control for histamine H1 and leukotriene CysLT1 receptors in qPCR and Western blot.

Fig. 5 presents the changes in the gene and protein expression levels before and after medication. The AR participants had significantly lower mRNA and protein expression levels of H1 and CysLT1 receptors after medication therapy than before treatment (*p* < 0.05), although no statistically significant difference (*p* > 0.05) in these levels was observed between the AR participants and healthy controls.

## Discussion

The main AR clinical classification method is based on the subjective clinical symptoms reported by patients with AR, such as nasal symptoms and quality of life. AR is classically subdivided into Seasonal AR (SAR), PAR, and mixed AR,^12^ although new classifications, that is, persistent and intermittent AR, were recently proposed.^13^ These classifications played a significant role in the development of AR treatment and prevention approach. However, these classifications mainly focus on subjective nasal symptoms reported by patients with AR, without considering the changes in the main inflammatory mediator levels. Hence, the severity of the disease and its effect on the quality of life of patients with AR are not objectively reflected. New, accurate, and objective clinical typing for AR is necessary for the development of effective, economical, and individualized treatment. AR has been reclassified as mild/moderate–severe and intermittent/persistent depending on the symptom duration based on the guidelines of Allergic Rhinitis and Its Impact on Asthma (ARIA)^5^ and those developed in China (2009 edition).^14^ This classification method takes into consideration the symptom onset and disease severity, which closely reflect the actual symptoms experienced by patients. Recently, the 2014 Practical Guideline for the Management of Allergic Rhinitis in Japan^15^ has reclassified AR into sneezing and nasal obstruction types based on the main nasal symptoms experienced by patients with AR depending on the allergy mediators, which may be more appropriate when determining the choice of clinical treatment.

In an attempt to provide a new, economical, and individualized treatment choice for patients with AR, we first combined the main nasal symptom evaluation with experimental detection of the changes in histamine and leukotriene D4 levels and their H1 and LT1 receptor expressions to explore the role of clinical typing and individualized treatment of AR. In this study, we divided all the participants into three groups based on the assessment results of the nasal symptoms and histamine and leukotriene D4 levels. Our classification depended on the combination of nasal symptom evaluation and experimental detection of histamine and leukotriene D4 levels to reflect the changes in the actual symptoms of the participants, which is a more reasonable and effective approach in determining the appropriate drug to be used than the previous methods.

Histamine and leukotriene D4 are the main inflammatory mediators that are implicated in AR pathogenesis. In a study by Vadas et al.^16^ that involved 41 participants who experienced acute allergic reactions, which ranged from Grade 1 (least severe) to Grade 3 (most severe), the proportion of participants with elevated histamine levels increased as the allergic reaction grade increase. A similar result was reflected in a study by Mayer et al.,^17^ which demonstrated that highly elevated histamine levels might serve as a sign for the development of fatal anaphylaxis. Changes in the leukotrienes levels during allergic reactions were similar to that of histamine. Additionally, obvious evidences exist suggesting that leukotrienes levels are increased in patients with AR following allergen exposure.^18^, ^19^, ^20^ Our results showed that the histamine and leukotriene D4 levels in the peripheral blood and nasal secretions of AR participants from all groups were consistently higher than those of healthy controls. Furthermore, the histamine and leukotriene D4 levels are highly associated with the changes in the sneezing and nasal obstruction symptoms, which is consistent with the results of previous studies.^18^, ^21^, ^22^ The elevation of the histamine level may be the cause for the increased vasodilation and vascular permeability of the nasal mucosal glands, which resulted in sneezing. On the other hand, the increase in the leukotriene D4 level may result in the dilation, hyperemia, and thickening of the nasal mucosa, which lead to nasal obstruction.

According to the guidelines of ARIA^5^ and those from China (2009 edition),^14^ the therapeutic agents for AR are classified into six categories: antihistamines, nasal steroids, leukotriene receptor antagonists, anti-cholinergic agents, mast cell stabilizers, and nasal decongestants. Out of all these therapeutic agents, second-generation antihistamines (loratadine) and nasal steroids are the classic first-line medications for AR treatment. Recently, leukotriene receptor antagonists (montelukast) have gained increasing attention in clinical research because of their effect on AR pathogenesis.^23^, ^24^ At present, many combination therapies, which include loratadine, montelukast, and nasal steroids, are proposed, and their specific clinical curative effects on AR have been evaluated.^6^, ^10^, ^25^, ^26^, ^27^ Our results suggested that loratadine and its combination clearly improved the sneezing symptom and significantly reduced the histamine level, whereas montelukast and its combination obviously improved the nasal obstruction symptom and significantly decreased the leukotriene D4 level. Our results are in agreement with the recent clinical practice guidelines for AR, which highly recommends the use of second-generation antihistamines with less sedating side effects in the treatment of AR patients with sneezing and nasal itching as the primary symptoms.^28^ The use of combination therapy is also recommended so that a “comprehensive” blocking effect is achieved as much as possible.

Histamine and leukotriene are considered as the principal inflammatory mediators with multiple physiological effects, which results from the binding of their receptors.^17^, ^29^ Histamine receptors are classified into four subtypes, namely, H1, H2, H3, and H4, which belong to the G-Protein Coupled Receptor (GPCR) family members.^30^ A study performed by Akdis et al.^31^ showed that H1 receptors are most critical in mediating histamine release in allergic disease. Cysteinyl Leukotriene (CysLts) receptors have three endogenous ligands, namely, Leukotriene C4, D4, and E4 (LTE4), which also belong to the GPCR family and are classified into two types (CysLT1 and cysteinyl leukotriene [CysLT2]) based on their sensitivity to CysLT1-selective antagonists.^32^ Previous studies demonstrated that CysLts have a clear role in the occurrence of pathophysiological conditions, such as asthma, AR, and other nasal allergies.^21^, ^33^, ^34^ A study by Figueroa et al.^35^ on the mRNA and protein expression of CysLT1 and CysLT2 receptors in inflammatory cells from nasal lavage samples collected from participants during active SAR demonstrated that CysLT1 receptor-positive cells were highly responsible for the development of inflammation, which indicates the potential of CysLT antagonists in treating both AR and asthma. In our study, AR participants had higher gene and protein expression levels of H1 and CysLT1 receptors than healthy controls. Additionally, significant reductions in the mRNA and protein levels of H1 and CysLT1 receptors were observed after 4 weeks of antihistamine and antileukotriene treatment, which is consistent with the changes in the relevant mediator levels. Notably, the gene and protein expression levels of AR participants were not statistically different (*p* > 0.05) from those of healthy controls after medication therapy. All these data suggest the efficacy of combination therapies, which significantly improved the nasal symptoms of participants with AR.

## Conclusions

In conclusion, clinical symptom evaluation combined with experimental detection of histamine and leukotriene levels is a more objective and accurate method to clinically classify AR than previous approaches. Furthermore, our results demonstrated that the use of combination therapies is effective and this finding opens new strategies for the individualized AR treatment.

## Funding

This work was supported by grants from Science and Technology Program Project of Guangdong Province (S2011010003947).

## Conflicts of interest

The authors declare no conflicts of interest.
